# Catastrophic health expenditure of cancer patients at the end-of-life: a retrospective observational study in China

**DOI:** 10.1186/s12904-019-0426-5

**Published:** 2019-05-23

**Authors:** Anli Leng, Jun Jing, Stephen Nicholas, Jian Wang

**Affiliations:** 1 Center for Health Economics Experiment and Public Policy, School of Public Health, Shandong University; Key Laboratory of Health Economics and Policy Research, NHFPC (Shandong University), Jinan, China. No. 44 Wenhuaxi Road, Lixia District, Jinan, 250012 China; 2 Jun Jing Research Center for Public Health, Medical School, Tsinghua University, Room B408, Beijing, 100084 China; 3 School of Economics and School of Management, Tianjin Normal University, West Bin Shui Avenue, Tianjin, 300074 China; 4 TOP Education Institute 1 Central Avenue Australian Technology Park, Eveleigh, Sydney, NSW 2015 Australia; 5 Research Institute of International Strategies, Guangdong University of Foreign Studies, Baiyun Avenue North, Guangzhou, 510420 People’s Republic of China; 6 Newcastle Business School, University of Newcastle, University Drive, Newcastle, NSW Australia; 7 Dong Fureng Institute of Economic and Social Development, Wuhan University, 54 Dongsi Lishi Hutong, Beijing, 100010 China

**Keywords:** Catastrophic health expenditure, Impoverishment, End-of-life, Cancer patients

## Abstract

**Background:**

Cancer is the second leading cause of death globally, causing a substantial economic burden on cancer suffers and their families. The aim of this study is to explore the prevalence, determinants and consequences of catastrophic health expenditure (CHE) among urban and rural end-of-life (EOF) cancer patients in China.

**Methods:**

Using respondent-driven sampling and face-to-face interviews, field research was conducted with a specialist questionnaire. Data were collected on 792 cancer patients who died between June 2013 and June 2016 in China. The determinants of household catastrophic expenditure were identified by multivariate logistic regression.

**Findings:**

It is found that more than 80% of cancer patients received life-extending treatment. Extremely high rates of CHE were identified among EOL cancer patients, at 94.3% for urban families and 96.1% for rural families. After spending for health, 84.1% of urban and 91.1% rural EOL cancer patient households were impoverished, falling below the poverty line. For both urban and rural households, income was the most significant factor associated with catastrophic health expenditure (CHE). Health insurance did not adequately compensate for CHE. Rural families experienced higher CHE, lower levels of health care utilization, a different mix of health care access and higher rates of borrowing for out-of-pocket (OOP) health care expenditures than urban families. Both urban and rural households suffered long-term economic disadvantage due to CHE and borrowing for OOP medical care expenses.

**Conclusions:**

EOL cancer patients experienced severe CHE, with families forced into poverty. With only about 1% of EOL cancer patients receiving palliative care, developing palliative care services and expanding the acceptance of palliative care in China is both urgent and essential. To help address impoverishment due to CHE, China should also develop targeted programs to reduce income inequality, especially rural-urban inequalities; increase access to health care; and accelerate health reform. Increasing the retirement age would provide households with more savings and wealth to withstand CHE.

## Introduction

Cancer is the leading cause of death in China [1]. The cancer care cost curve displays a distinctive U-shape distribution, with the most resource-intensive stage of health care occurring at the end of life (EOL). The EOL period marks the highest levels of hospital care and the point where medical costs strain family income and household savings the most. For example, one-third of all direct medical costs of cancer treatment in the United States occurred in the final year of life, with approximately 80% of that expenditure occurring in the last month of care [2]. While cancer care imposes strains on the financial resources of all households, the heavy financial burden during the EOL period means some cancer households incur catastrophic health expenditures (CHE) [3]. How heavy is the economic burden on cancer patients and their families in China? Surprisingly, there are few EOL studies of cancer patients in China, and even fewer studies of CHE. This paper explores the prevalence, determinants and consequences of catastrophic health expenditure among urban and rural cancer patients in China in their last 3 months of life.

Catastrophic health expenditure (CHE) is out-of-pocket (OOP) medical expenses in access of 40% of household income, after any government health care subsidy, which reduces non-health household expenditure below the level required for necessities [4]. CHE on chronic diseases like cancer [5, 6], diabetes [7], cardiovascular diseases [8], hypertension [9] and mental health [10] means a heavy economic burden on individuals and families, pushing many households into poverty [11–14]. The evidence shows that the economic burden of CHE was linked to health care utilization [15], especially hospital care [16, 17], which was not off-set by health insurance [18–21]. Household economic status was a key determinant of the ability of families to meet OOP expenditures [16, 17, 20]. A study of eight southeast Asia countries found low income category families faced significantly higher odds of catastrophic health expenditure than better off families who had higher wages and larger family financial savings [15, 22]. The rural-urban income inequality divide also meant rural households will less able to pay for OOP health costs than urban households. For example, Turkish rural households faced a 2.5 times greater exposure to CHE than urban residents [23].

We expect that cancer-related CHE to be related to wealth inequalities and the dual urban-rural economic structure in China. China’s severe wealth inequalities, with the richest 1% owning more than 33% of the total national household wealth, while the poorest 25% owned less than 2% of the wealth [23], made poorer Chinese households especially vulnerable to CHE. Further, poor Chinese families were more likely to live in rural areas, and China’s dual urban-rural economic structure meant rural households suffered disparities in income, age structure and provision of health care compared to urban households [12, 24]. For example, there were significant urban-rural differences in access to health insurance. Before 2016, the New Rural Cooperative Medical Scheme (NCMS), Medical Insurance for Urban Residents scheme (MIUR) and Medical Insurance for Urban Employees scheme (MIUE) had different deductibles, limit lines and reimbursement rates, with the MIUE offering the highest reimbursement rate (55%), followed by the MIUR (40%) and NCMS (30%) [11].

In this article, we explore the prevalence, determinants and consequences of catastrophic health expenditure (CHE) among urban and rural end-of-life (EOF) cancer patients in China.

## Methods

### Study population

Data are on patients during the last three months of life, who suffered a cancer disease according to the International Statistical Classification of Diseases and Related Health Problems, Tenth Revision (ICD-10), cancer diagnosis codes and had died between June 2013 and June 2016. Drawn mainly from Shandong (47.6%), Shan’xi (11.24%), Yunnan (9.47%), Jiangxi 7.83%), Henan (5.30%), Anhui (4.92) provinces, with the remainder other provinces, autonomous regions and municipalities (13.64%), the sample comprised 792 advanced cancer patients, suffering from five main types of cancer: lung, intestinal, gastric, liver, and esophagus cancer.

### Data collection

Deceased cancer patients are an archetypical hidden population, and families of deceased cancer were also a hard-to-reach population, especially given China’s social norms that made families reluctant to publicly acknowledge their relatives’ cancer status. Alternative data on deceased cancer patients, such as hospital and insurance records, were strictly limited by confidentiality and privacy. To sample such a hard-to-reach population, we used respondent-driven sampling (RDS), which uses social chain referrals in the target population’s social network to collect personal health related data [25–28].

Twenty field research trained medical students sought support from the health care professionals in provincial health care facilities to establish contact with caregivers and relatives of deceased cancer patients. Based on the demographic characteristics of the population-based cancer deaths in China, 12 rural and 8 urban “seeds” of the target population comprised wave 1 of a rigorous chain recruitment process, where seeds were given coupons to recruit success waves of respondents [25–27] Providing unbiased population estimates [25–27], RDS mitigates bias in the sample’s socio-demographic characteristics, by recruiting caregivers of deceased cancer patients reflecting the urban and rural location and different socio-economic backgrounds, marital status and ages of China’s population-wide deceased cancer patients. All surveys were conducted face-to-face, where the primary caregiver was asked detailed questions about the deceased cancer patients and coupons were used to gain referrals to other caregivers, who can remain anonymous, if desired [29]. Data collection continued for two months, with a maximum chain length of six waves, allowing an equilibrium sample size on the key variables to be achieved. Previous studies reported that valid and representative samples could be obtained by six or more RDS waves [28]. Importantly, we found no statistically significant differences in socio-demographic characteristics between our sample and population-based cancer deaths in China [30–32].

The deceased patient’s demographic characteristics, health services utilization and EOL out-of-pocket payments were included in the questionnaire. The interviews provided a retrospective database, including health service expenses related to the patient’s cancer treatment and related health cost issues. While one challenge of retrospective surveys is recall bias, there are a number of reasons for confidence in our data, especially the accuracy of the health care expenditure information. Reimbursement procedures were complex, with patients and their carers paying medical expenses before seeking reimbursement from their medical insurance, which meant medical expenses were recalled from the initial payment, then again as part of the health insurance claims. Medical insurance claim forms required accurate information. Second, OOP expenses accounted for a significant proportion of family income and savings, and these large demands on savings and income would have been a matter of ongoing and constant concern. Frequently, family and friends were called on for loans to pay for the cancer patient’s medical costs, another reason to be confident that the medical costs were accurate recalled. The interviewers were trained to prompt interviewees to carefully recollect medical expenses. When caregivers were uncertain about OOP expenses, additional information was sought from the patient’s family doctor and, when available, patients’ medical records were used to identify some medical costs and invoices were used to calculate expenses.

### Key variables

Our data comprised information on the patient’s gender, age, marital status, residence, occupation status, monthly household income and expenditures, health insurance status, cancer site and health care utilization, including impatient and outpatient care. Catastrophic health expenditure was defined as OOP health payments exceeding 40% of a household’s income [11]. The impoverishment rate referred to whether OOP cancer health-care payments caused the household to drop below the poverty line [33]. Rural poverty was defined by the State Council Leading Group Office of Poverty Alleviation and Development in China as below RMB2300 per capita annual income [34]. There is no unified national urban poverty line. To measure urban poverty, we used a urban poverty line calculated by Chen et al. which was RMB4500 per capita annual income [35].

### Statistical analysis

The proportion of households with catastrophic medical expenditure and the household impoverishment rate were calculated. Descriptive statistics were used to compare the distribution of patient social-demographic characteristics, health care utilization and OOP expenditure at the end of life by gender and geographic locations. Logistic regression was used to analyze the determinants of catastrophic health expenditure [33]. All costs were adjusted to 2016 prices. The analyses were conducted using STATA, version 14 and significance was assessed at level of 0.05.

## Results

### Characteristics of the study population

Table 1 presents the distribution of social-demographic characteristics of EOL cancer patients between urban and rural areas. There were no significant urban-rural differences by age (about 50% were over 65) and marital status (roughly 80% married). However, there were important urban-rural differences: compared with urban patients, rural patients were more likely to be male, less likely to have steady work and more likely to have a shorter survival time from diagnosis to death. The main insurance plan of rural patients was NUMS, while urban patients were insured mainly under the MIUE.

**Table 1:** Characteristics of the study sample by geographical location (*N* = 792)

Characteristics	Urban area (*n* = 195)			Rural area (*n* = 597)			*p* value*
	N	%	Mean	n	%	Mean	
Gender			NA			NA	0.003
Male	116	59.49		423	70.85		
Female	79	40.51		174	29.15		
Age			64.75			64.01	0.588
Age < 45	15	7.69		44	7.49		
Age 45–55	37	18.97		77	13.12		
Age 55–65	40	20.51		174	29.64		
Age 65–75	48	24.62		169	28.79		
Age ≥ 75	55	28.21		133	22.66		
Marriages							
Unmarried	39	20.00		96	16.08		0.206
Married	156	80.00		501	83.92		
Occupation							0.000
Farmer	37	18.97		476	79.73		
Steady workers	27	13.85		42	7.04		
Non-regular workers	55	28.21		55	9.21		
The retired	76	38.97		24	4.02		
Insurance status							0.000
MIUE	92	47.18		37	6.20		
MIUR	28	14.36		37	6.20		
NUMS	37	18.97		473	79.23		
Other insurance plan	30	15.39		21	3.52		
None	8	4.10		29	4.85		
Days from diagnosis to death			548.82			448.29	0.000
≤ 3 months	35	17.95		96	16.08		
3 months–6 months	33	16.92		123	20.60		
6 months-1 year	57	29.23		157	26.30		
1 year-2 years	40	20.51		137	22.95		
≥ 2 years	30	15.38		84	14.07		
Cancer site							0.000
Lung	42	21.54		145	24.29		
Intestinal	18	9.23		35	5.86		
Gastric	27	13.85		99	16.58		
Liver	30	15.38		105	17.59		
Esophagus cancer	8	4.1		78	13.07		
Other	70	35.9		135	22.61		

*p-value from Chi-squared test or T-tests

### Distribution of household income quintile by rural and urban areas

Table 2 shows the quintile distribution of household income by rural and urban areas. We divided urban and rural patients into five income groups based on the monthly household income, ranging from the poorest (1st quintile) to the richest (5th quintile) households. The average household income in urban areas was roughly three times that of rural areas.

**Table 2:** Distribution of household income quintile by rural and urban areas (N = 792)

Monthly household income	Urban area (n = 195)			Rural area (n = 597)		
	N	%	Mean (US$^b^)	n	%	Mean (US$)
Quintile 1^a^	42	21.54	88.45	150	25.13	29.73
Quintile 2	40	20.51	287.30	93	15.58	75.75
Quintile 3	35	17.95	524.24	125	20.94	142.87
Quintile 4	44	22.56	882.49	112	18.76	276.85
Quintile 5	34	17.44	2127.63	117	19.60	879.69
Total	195	100.00	742.17	597	100.00	273.52

*US$* United States dollars

^a^Quintile 1 is the poorest and quintile 5 is the wealthiest

^b^Based on a currency exchange rate of the 6.6423 yuan to US$1.00 in 2016

### Health care services utilization

**Fig. 1:**
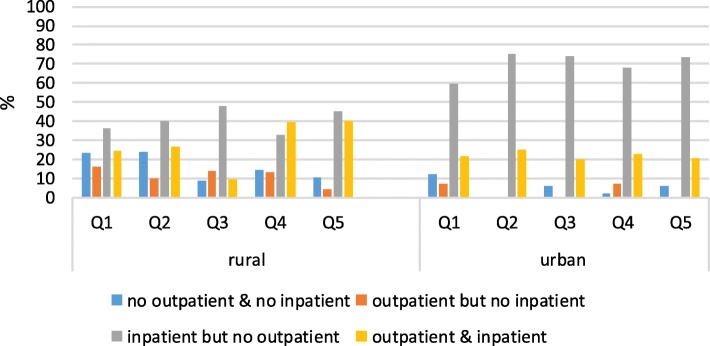
Urban-rural disparities of health care utilization through income quintile (*N* = 792)^a^ Q1 is the poorest and Q5 is the wealthiest

Figure 1 presents urban-rural disparities in health care utilization by income quintiles. Rural patients at the end-of-life were significantly less likely to access inpatient care than urban patients, who had high inpatient care rates across all income quintiles. Rural patients were also more likely to mix outpatient and inpatient care compared to EOL urban patients who overwhelmingly opted for only inpatient care. Patients in the lowest two rural income quintile were the least likely to access health care (about 20%); the least likely to access inpatient care (about 35%); and the most likely to use only outpatient services (about 15%) compared to all the other income quintile. Among 792 decedents, only 9 (1.14%) cancer patients received hospital palliative care at the end of life, of which 4 were urban cancer patients and 5 rural patients.

### EOL health expenditure and out-of-pocket expenditure by quintile

**Fig. 2:**
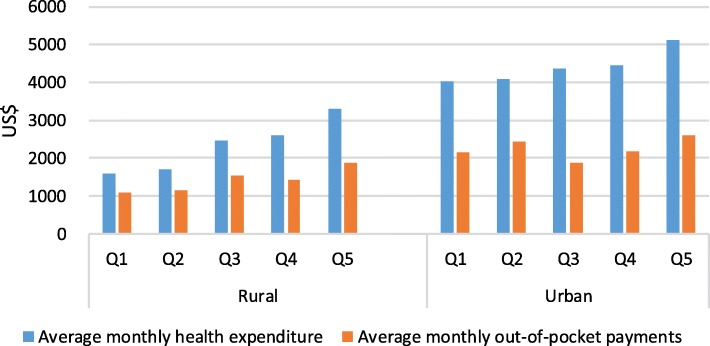
Monthly health expenditure and out-of-pocket payments through income quintile (N = 792) US$, United States dollars.^a^ Q1 is the poorest and Q5 is the wealthiest.^b^ Based on a currency exchange rate of the 6.6423 yuan to US$1.00 in 2016

Figure 2 reports that the average EOL health expenditure and EOL out-of-pocket expenditure of urban cancer patients were about two times that of rural patients. But calculating the share of monthly health expenditure and OOP expenditure in family income, rural patients allocated a higher proportion of household income to health care and to OOP expenditures. The proportion of family income allocated to health expenditures and OOP expenses was higher for low income quintiles than for high income quintiles.

### Catastrophic health expenditure by quintile

Table 3 shows that 94.3% of urban households and 96.1% of rural households spent 40% or more of their monthly income as out-of-pocket cancer health care expenditure, signaling very high levels of CHE at the end of life. The poorer the household, the higher the prevalence of catastrophic health expenditure. OOP payments were about 24 times household income in the urban poorest income quintile, reaching up to about 36 times household income in the lowest rural income quintile.

**Table 3:** Urban-rural disparities of the prevalence of catastrophic health expenditure through income quintile (N = 792)

Variable	Rural (n = 597)						Urban (n = 195)						*p* value*
	Q1	Q2	Q3	Q4	Q5	total	Q1	Q2	Q3	Q4	Q5	total	
OOP payments share in total health expenditure (%)	68.09	68.82	62.27	54.75	56.94	60.72	53.21	43.33	42.74	49.13	42.48	46.39	0.000
OOP payments share in household income (%)	3635.62	1500.00	1069.38	513.19	214.20	513.41	2423.21	614.95	356.78	248.08	102.25	273.92	0.000
Households with catastrophic health expenditure (%)	100	100	98.40	94.60	87.20	96.10	100	100	100	95.50	73.50	94.30	0.285

^a^Q1 is the poorest and Q5 is the wealthiest

^*^p-value from Chi-squared test

### Poverty levels and impoverishment

**Fig. 3:**
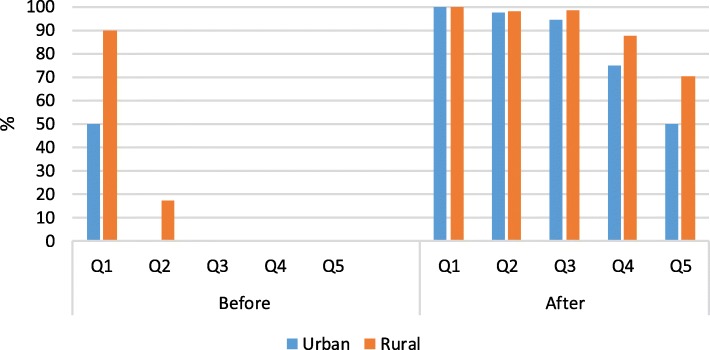
The proportion of poor households before and after out-of-pocket payments (N = 792).^a^ Q1 is the poorest and Q5 is the wealthiest

Before any out-of-pocket health care expenditures, poor households accounted for 10.8% of all urban households. But 50% of urban households in the lowest quintile fell below the poverty line. (see Fig. 3). In rural areas, poor households accounted for 25.3% of all rural households. But 90% of rural households in the first quintile and 17.2% in the second quintile fell below the poverty line. After OOP spending for EOL cancer care, 84.1% of all urban households and 91.1% of all rural households became impoverished, falling below the urban or rural poverty line. Impoverished households comprised 100% in the first income quintile, irrespective of urban or rural location.

### Borrowing money to undertake the catastrophic out-of-pocket payments

**Fig. 4:**
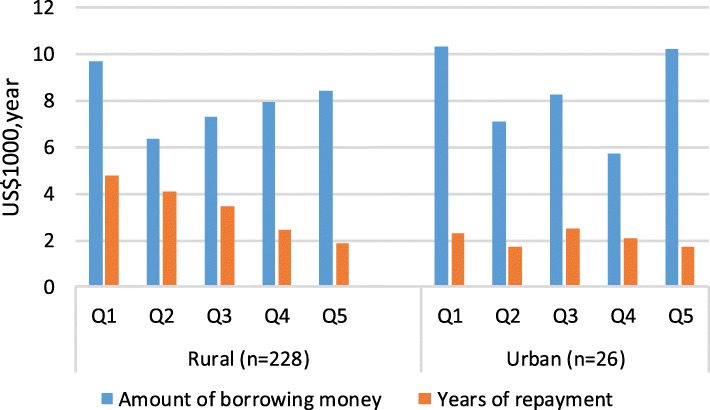
Borrowing money from relatives and friends among catastrophic households (N = 792) US$,United States dollars.^a^ Q1 is the poorest and Q5 is the wealthiest.^b^ Based on a currency exchange rate of the 6.6423 yuan to US$1.00 in 2016

Figure 4 reveals that 254 households, roughly one-third of the sample, borrowed money from their relatives and friends to meet cancer-related EOL medical costs. Irrespective of urban-rural location, the largest amounts of money borrowed, roughly US$10000, were by the poorest income quintile households, followed by the richest income quintile households. The average repay time was inversely related to the income quintiles, and much longer for rural households than urban households.

### Determinants of catastrophic health expenditure

Table 4 presents estimates of the determinants of catastrophic health expenditure. Household income was the most significant factor associated with catastrophic health expenditure. Households with higher income were less likely to incur catastrophic health expenditure no matter their urban or rural location. In contrast to urban households, rural patients receiving inpatient services and outpatient services significantly increased their risk of catastrophic out of pocket expenses.

**Table 4:** Determinants of catastrophic health expenditure in logistic regression model (N = 792)

Variable	Urban			Rural		
	OR	95%CI	*p* value	OR	95%CI	*p* value*
Age	3.09	0.43–22.47	0.264	0.66	0.24–1.84	0.431
Gender	1.11	0.18–6.92	0.910	1.19	0.38–3.74	0.765
Monthly household income	0.12	0.03–0.55	0.006	0.24	0.12–0.46	0.000
Days from diagnosis to death	0.60	0.29–1.22	0.159	1.04	0.67–1.62	0.857
Inpatient	0.33	0.02–6.93	0.478	12.82	4.29–38.32	0.000
Outpatient	1.23	0.17–8.72	0.834	3.11	1.00–9.64	0.049
Insurance	0.83	0.47–1.46	0.523	1.04	0.72–1.52	0.822

*p-value from logistic regression model

## Discussion

Our study found that out-of-pocket payments for EOL cancer patients meant 94.3% of urban and 96.1% of rural patients incurred CHE. These proportions are much higher than that reported in other countries, with CHE rates significantly lower in Malaysia (47.8%) [32], South Korea (39.8%) [36], Iran (67.9%) [6] and eight Southeast Asian countries (48%) [15]. Even more concerning, 84.10% urban EOL cancer households and 91.10% rural EOL cancer households in China dropped below the poverty line after out-of-pocket payments. These rates of EOL cancer patient impoverishment contrast with 7.5% of all Chinese households (not EOL cancer households) officially in poverty in the Fourth National Health Service Survey [11]. In China, out-of-pocket payments of EOL cancer patients brought impoverishment to a significant proportion of lower income families, especially rural households, and imposed a serious economic burden to most households irrespective of income or urban-rural location.

The key role of household income in catastrophic health expenditure was consistent with studies in other countries [13, 17, 20, 32]. In China, poor households and rural households were the most likely to face impoverishment and economic hardship. Income inequality exacerbated the hardships of catastrophic out-of-pocket payments. As shown in Table 3, the share of catastrophic OOP payments in household income in the poorest quintile was about 17 times that in the richest quintile in rural areas and 24 times that in urban area. To pay for catastrophic OOP expenditures, families, especially in the lower income quintiles, borrowed from their relatives and friends, with poorer household requiring more time for loan repayments than richer households. With between two and five years required for loan repayment, CHE not only pushed families into poverty, but created a long-term household economic burden.

We also found the health care utilization was another determinant of CHE for EOL cancer patients, especially in rural households, which is also consistent with other studies [15, 16]. Rural patients were more likely to be outpatients, while urban patients were more likely to be inpatients. This reflected, in part, rural-urban differences in inpatient and outpatient care, with rural patients accessing village and township clinics and county hospitals, while urban patients accessed city and provincial hospitals. Our sample did not contain observations from remote areas, with both rural and urban patients in our sample accessing health facilities within 5 km of their home [37]. Households with hospitalized patients were 12.8 times more likely to incur catastrophic health expenditure than households with outpatient care; households accessing outpatient care were 3.1 times more likely to incur CHE than patients not accessing health care. Potential OOP expenditure may have discouraged inpatient care and encouraged patients to seek either outpatient care or no health care.

Health care insurance had a limited impact on OOP health care expenditures. The average insurance reimbursement rate for cancer patients was only 40% in rural households and 54% in urban households, which helps explain why health insurance was not a significant factor explain CHE in our study. Further, the urban-rural differences in NCMS, MIUE and MIUR reimbursement rates meant insured rural household bore higher out-of-pocket expenditures than insured urban households, which imposed a higher economic burden on rural families and reduced their access to health care facilities relative to urban households. For example, the poorest households in the rural area suffered the most severe economic burden. The out-of-pocket EOL cancer care expenses were about 36 times rural household income, incurring borrowings of up US$10,000 from their relatives and friends, with about a five year pay-back period.

While the urban-rural rates of catastrophic health expenditure were not significantly different from each other, they were both extremely high. Further, catastrophic out-of-pocket payments by EOL cancer patients imposed a much higher economic burden on rural than urban households. The poorest households suffered a greater economic burden than rich families from CHE, and rural households suffered greater OOP expenses, and a more severe economic burden, than urban families.

Addressing the CHE challenges to families requires an equitable, affordable and cost-effective people-centered health care system, dependent on deepening health care reform in China [38]. *Healthy China 2030* outlines an ambitious reform program, including universal health insurance coverage, a national essential medicines system, improvements in the primary care system, equitable public health services access and public hospital reform [39]. Enhancing China’s primary health-care system and reform of public hospitals will both reduce medical costs and improve equity and access to health care. These reforms are particularly urgent for rural patients. Expanding the scope and depth of health insurance and reducing the co-payment for inpatient services provides a direct way to reduce the out-of-pocket medical payments. *Healthy China 2030* identifies the need to develop a multi-tiered medical insurance system, with basic medical insurance as the main body, but various other forms of supplementary insurance and commercial health insurance as supplements [39]. Catastrophic health insurance would provide a supplementary health insurance plan, but it would need to be structured to ensure widespread availability, especially to poorer and rural families.

We also recommend investing in palliative care facilities. We found that among 792 decedents, only 1.14% of cancer patients received hospital palliative care at the end of life. The proportion of cancer patients receiving palliative care in the United States was about 40 times greater than in China, where about 46% patients received at least one-day palliative care [40]. Poorly serviced by palliative care, which provides patients with alternative medical treatment choices, Government action is required to increase the size of palliative medicine-trained workforce and promote optimal mixes of public-private palliative care, especially for poorer rural communities. Besides investing in palliative care facilities, there are additional challenges facing palliative care in China. First, there is a long way to go for improving the acceptance and popularity of palliative care. Second, the insurance schemes need to provide adequate reimbursement for palliative care, which will also be conducive to improve the acceptance of palliative care. Finally, adequate education and perception of palliative care as end-of-life care should be promoted [1].

More generally, educating EOL patients on making appropriate health care choices would help to avoid overtreatment, especially reducing inpatient care for urban patients. It is unclear how families make EOL treatment decisions, and we speculate that treatment choices will vary between rural and more traditional family value families and urban families. Education and training will also help health care professionals to identify patients who should not receive treatment or receive outpatient rather than inpatient treatment or be directed to palliative care. Therefore, education programs that seek to change culture-based decision-making by EOL patients, EOL patients’ families and health professionals need to be modeled for different cultural and urban-rural groups in China.

Considering that average age of EOL cancer patients in our study was about 64 years old, encouraging longer periods of work would improve the ability to pay OOP catastrophic health costs. By international standards, China’s retirement age is low, on average 60 years old for men and 55 for women. With China’s aging population, postponing retirement ages will enhance the earning capacity and wealth of families, and also ease the old-age burden on the medical system. Further, government promoted labor income plans at the province, city and county level, such as organizing farmers to seek non-agricultural work and enter business, might be extended to medical assistance programs to cover out-of-pocket payments, especially for rural households. Finally, social security schemes and poverty alleviation programs could be accelerated to lift families out of poverty, caused by CHE [41].

## Limitations

There are a number of limitations in our study. First, we obtained OOP expenses through retrospective data reported by the caregivers. In spite of impact on family income and savings of OOP expenses, their payment to hospitals before being reclaimed as part of insurance reimbursements, the borrowing from relatives and friends to cover medical expenses by many families and the training of interviewers to prompt accurate information, some OOP cost data might be missing or inaccurate. Second, we calculate the impoverishment of households based on their current income, not household savings and wealth. This could overestimate the proportion of families impoverished by CHE. However, when out-of-pocket payments are financed by savings, depleting household assets, households are vulnerable to future shocks on household resources and to future impoverishment. Our study found that borrowing to pay for OOP medical expenses put households at risk, imposing a long-term financial burden. Third, we only analyze the out-of-pocket spending of EOL cancer patients. If there are other family member suffering chronic diseases, the proportion of households facing catastrophic health expenditure might be higher than that in our study. These limitations need to be taken into consideration in further studies, including collecting additional data on treatment days, hospital stays and cancer stages.

## Conclusions

Out-of-pocket health payments of EOL cancer patients were financially catastrophic for most rural and urban households, pushing families into poverty. The economic status of households and their health care utilization were significant factors associated with the CHE. Lower income and rural EOL cancer patients experienced severe CHE, with households forced into poverty. To address CHE, the government must improve the development of palliative care services and expand the acceptance of palliative care. In addition, accelerating health reform, educating medical professionals to offer and patients to understand alternative EOL medical treatment programs is vital. Providing a range of supplementary health insurance options, especially increasing adequate reimbursement for catastrophic health insurance and establish institutions to provide palliative care services, would provide vulnerable families a safety net during catastrophic illnesses. There were significant differences in rural-urban health care access, health insurance rebates and income levels that influenced the likelihood of impoverishment for households facing CHE. Better targeted programs to deal with rural poverty and income inequalities are required. Increasing the retirement age would help address savings and wealth levels of rural and poor urban households.

## Abbreviations

CHE: Catastrophic Health Expenditure
EOF: End-of-life
ICD-10: the International Statistical Classification of Diseases and Related Health Problems Tenth Revision
MIUE: Medical Insurance for Urban Employees Scheme
MIUR: Medical Insurance for Urban Residents Scheme
NCMS: New Rural Cooperative Medical Scheme
OOP: Out-of-pocket
RDS: Respondent-driven sampling
